# Implementation of Nurse-Led, Goal-Directed Lung Physiotherapy for Older Patients With Sepsis and Pneumonia in the ICU

**DOI:** 10.3389/fmed.2021.753620

**Published:** 2021-11-22

**Authors:** Jianhua Sun, Na Cui, Wen Han, Qi Li, Hao Wang, Zunzhu Li, Wei Cheng, Hongbo Luo, Mingxi Zhao

**Affiliations:** ^1^Department of Critical Care Medicine, State Key Laboratory of Complex Severe and Rare Diseases, Peking Union Medical College Hospital, Peking Union Medical College, Chinese Academy of Medical Science, Beijing, China; ^2^Department of Critical Care Medicine, Beijing Jishuitan Hospital, Beijing, China

**Keywords:** goal-directed lung physiotherapy, older patients, sepsis, pneumonia, intensive care unit

## Abstract

**Objectives:** This study aimed to investigate the effect of nurse-led, goal-directed lung physiotherapy (GDLPT) on the prognosis of older patients with sepsis caused by pneumonia in the intensive care unit.

**Methods:** We conducted a prospective, two-phase (before-and-after) study over 3 years called the GDLPT study. All patients received standard lung therapy for sepsis caused by pneumonia and patients in phase 2 also received GDLPT. In this study, 253 older patients (age ≥ 65 years) with sepsis and pneumonia were retrospectively analyzed. The main outcome was 28 day mortality.

**Results:** Among 742 patients with sepsis, 253 older patients with pneumonia were divided into the control group and the treatment group. Patients in the treatment group had a significantly shorter duration of mechanical ventilation [5 (4, 6) vs. 5 (4, 8) days; *P* = 0.045], and a lower risk of intensive care unit (ICU) mortality [14.5% (24/166) vs. 28.7% (25/87); *P* = 0.008] and 28 day mortality [15.1% (25/166) vs. 31% (27/87); *P* = 0.005] compared with those in the control group. GDLPT was an independent risk factor for 28 day mortality [odds ratio (OR), 0.379; 95% confidence interval (CI), 0.187–0.766; *P* = 0.007].

**Conclusions:** Nurse-led GDLPT shortens the duration of mechanical ventilation, decreases ICU and 28-day mortality, and improves the prognosis of older patients with sepsis and pneumonia in the ICU.

## Introduction

Aging of the population is a critical worldwide trend. The proportion of individuals older than 60 years has tripled over the last 50 years and will triple again before 2050. This aging has major consequences on the health system, including the intensive care unit (ICU). In the USA, almost half (48.7%) of the patients admitted to an ICU are aged 65 years or older, and patients aged 85 years or older account for 7 to 25% of the admission rate (1, 2). The rate of pneumonia increases rapidly with age. Approximately 19% of adults hospitalized with pneumonia have an ICU admission, including 60.7% of patients aged 65 years or older (3). Pneumonia in older patients can lead to cardiopulmonary failure, sepsis, and even systemic multiple organ failure, and they have a high mortality rate (4). Older people with pneumonia are at risk for a longer hospital stay, extended antibiotic therapy, and higher healthcare costs (5, 6). Therefore, an appropriate treatment protocol for pneumonia in older patients needs to be determined.

Because of age-related changes in the body, comorbidity, and malnutrition, the onset of pneumonia is insidious, rapid, and critical, and clinical treatment is difficult in older patients (2, 7, 8). Lung physiotherapy plays an important role in treatment of older patients with pneumonia (2, 7, 8). Lung physiotherapy can help patients in reducing the accumulation of airway secretion, clearing the respiratory tract, preventing a collapsed lung, improving lung compliance, and reducing comorbidities (9, 10). Nurses are responsible for most respiratory treatment in China. However, there is little evidence for the effect of nurse-led GDLPT on the prognosis of pneumonia in older patients with sepsis. In a previous study, we found that nurse-led GDLPT improved the outcomes in patients with sepsis and pneumonia (11). In this study, we carried out a retrospective analysis of older patients (age ≥ 65 years) with sepsis and pneumonia to evaluate whether GDLPT affects the clinical outcome.

## Materials and Methods

### Study Design and Patients

The GDLPT study was a prospective, two-phase (before-and-after) study conducted in Peking Union Medical College Hospital of China from January 2017 to January 2020. Details of this study, including the inclusion and exclusion criteria, have been published previously (11). The study period was divided into two stages of phase 1 and phase 2. During phase 1 (January to December 2017), patients received standard physiotherapy for pneumonia, and patients in phase 2 (February 2018 to January 2020) also received GDLPT. The two study periods were separated by a 1 month washout period that was dedicated to teaching the GDLPT protocol to all nursing and medical staff. The study protocol was approved by the ethics committee of Peking Union Medical College Hospital (approval number: JS-1170). All family members agreed to participating in the study and signed an informed consent form, and the study was registered at chictr.org.cn (identifier: ChiCTR-ROC-17010750).

**Figure 1:**
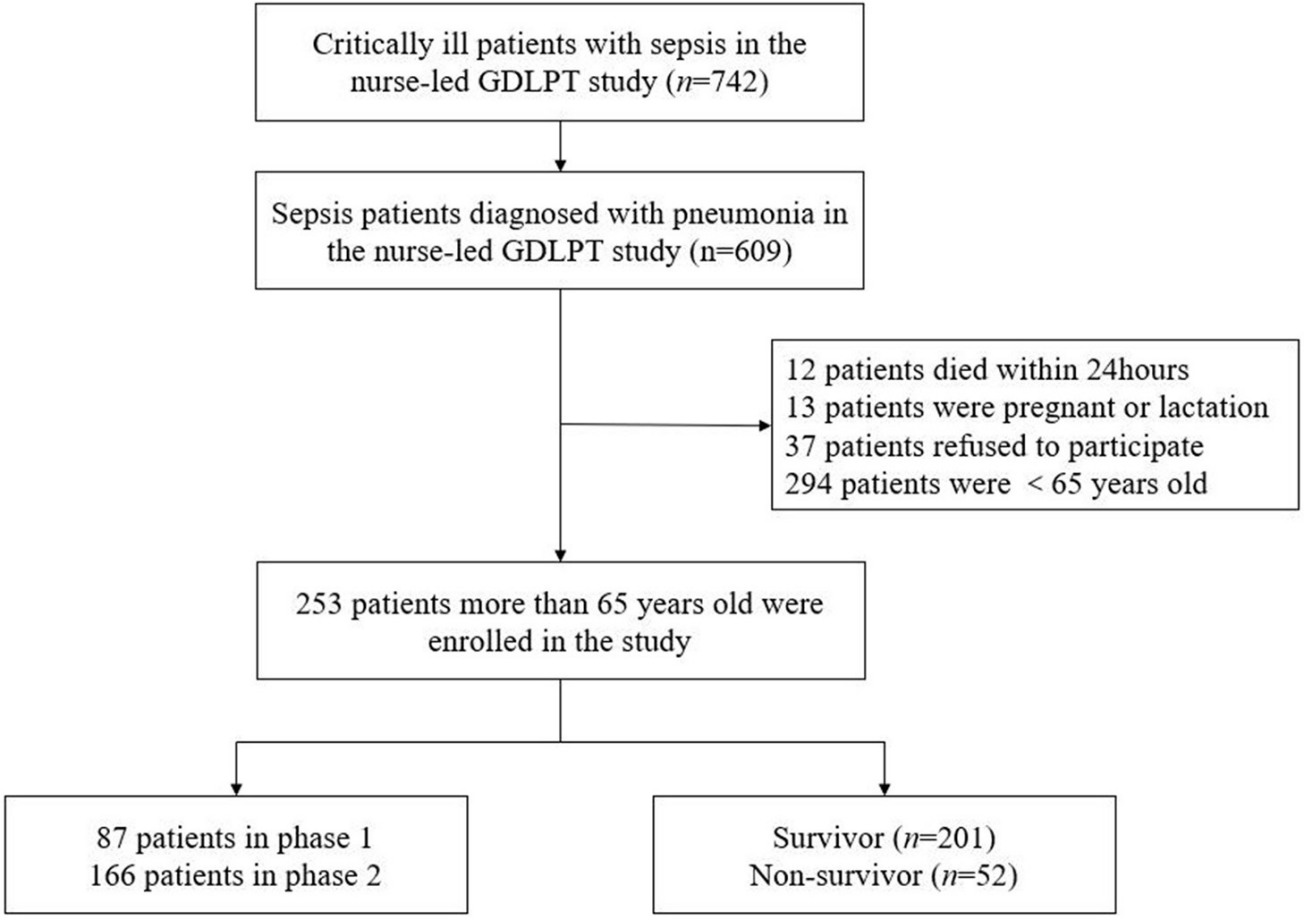
The flowchart of patients screening and selection in this study.

Figure 1 shows a flow diagram of patient screening and selection for this study. In the nurse-led GDLPT study, 609 of 742 patients were diagnosed with sepsis caused by pneumonia, of whom 253 (41.5%) were older than 65 years. Demographics, comorbidities, clinical parameters, and laboratory data were analyzed.

### Intervention and Comparisons

**Figure 2:**
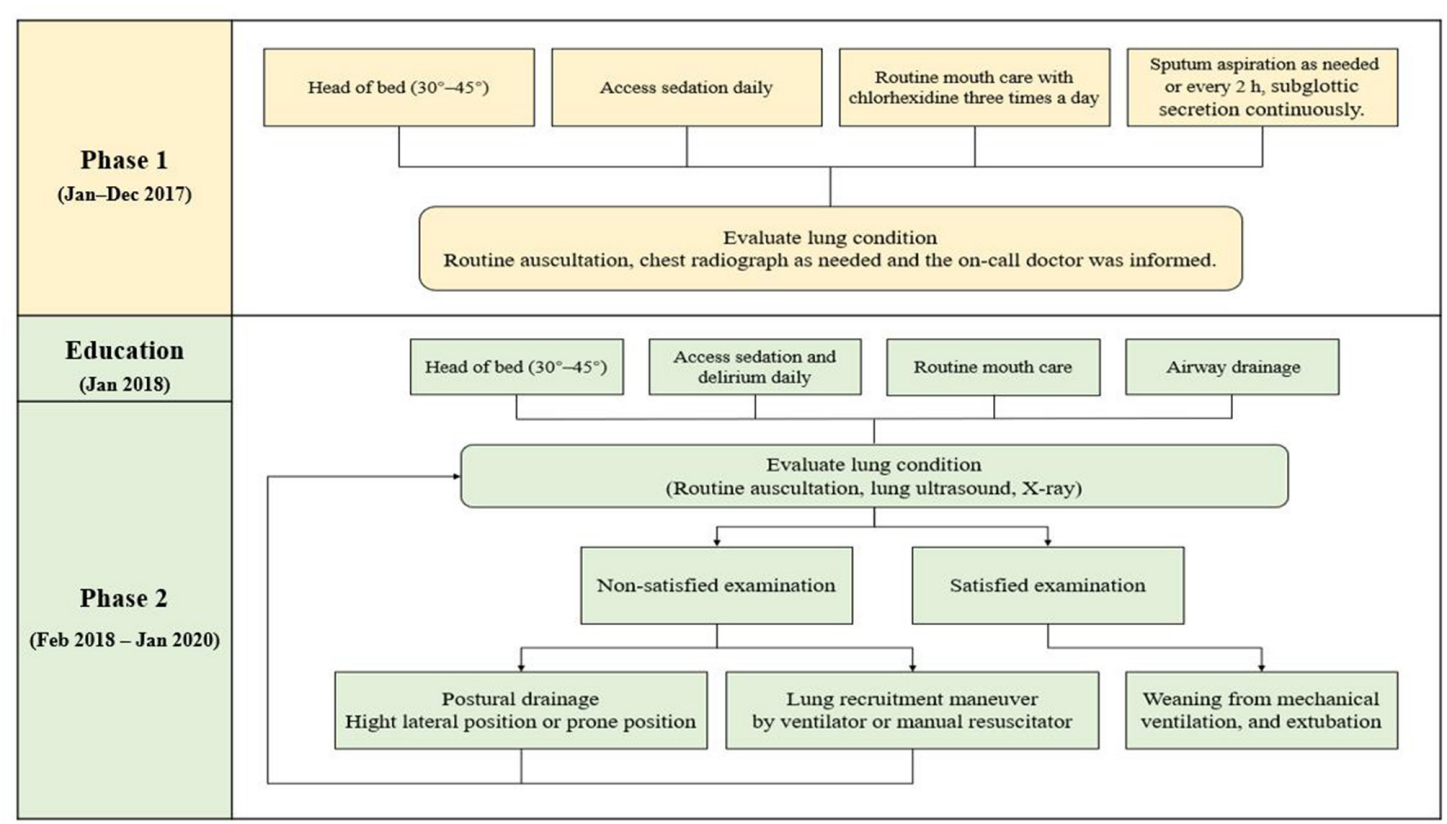
The protocols for pneumonia in the pre- and post-protocol groups.

The protocols for pneumonia in the pre- and post-protocol groups are shown in Figure 2. Details of this study, including study details such as the intervention objective, methods, and intervention frequency, were the same as those in our previous study (11). The Supplementary File provides additional evidence relevant to the intervention and comparisons.

### Data Collection

Baseline data of the patients, including sex, age, underlying diseases, the Sequential Organ Failure Assessment (SOFA) score, the Acute Physiology and Chronic Health Evaluation (APACHE) II score, and the Clinical Pulmonary Infection Score (CPIS), were analyzed. Vital signs, laboratory parameters, arterial blood gases, ventilatory parameters, life-sustaining treatments, and infection-related data during admission were also included. The data used in this study were the worst values in the first 24 h after ICU admission. The primary outcomes of this study were 28 day mortality. The secondary outcomes were the duration of ventilation, the length of the ICU stay, and the ICU mortality rate.

### Statistical Analysis

Data were analyzed by IBM SPSS version 21.0 (IBM Corp., Armonk, NY, USA). The frequency and percentage and mean and standard deviation were calculated for descriptive statistics. Bivariate analysis was performed using the chi-square test for categorical variables and the *t* test or one-way analysis of variance for continuous variables. Logistic regression analysis was performed with 28 day mortality as the dependent factor, which was significant (*p* < 0.2) in univariate analysis.

## Results

### Baseline Characteristics

Among 742 patients with sepsis, 253 patients who were older than 65 years were included in this study. Table 1 shows the clinical characteristics of the patients who were enrolled in this study. The median age of the patients was 72 years (interquartile range: 68–78 years) and 62.1% (157/253) were men. At admission to the ICU, the mean SOFA score was 11.49 ± 3.95, the median APACHE II score was 18 (12–18), and the median CPIS score was 6 (5–8). Ninety-two (36.4%) patients had heart failure, 19 (7.5%) had chronic obstructive pulmonary disease, 74 (29.2%) had diabetes mellitus, 3 (1.2%) had liver cirrhosis, 44(17.4%) with tumors, and 18 (7.1%) had chronic renal failure. There were no significant differences in age, sex, underlying diseases, source of admission, disease severity scores (APACHE II, SOFA, and CPIS scores), vital signs (heart rate, respiratory rate, and mean arterial pressure), ventilatory parameters, arterial blood gases, clinical laboratory evaluation, life-sustaining treatments, coexisting pathogens, or drug therapy between the two groups after ICU admission.

**Table 1:** Characteristics of patients in the control and treatment groups at ICU admission.

**Variables**	**Sepsis (*n* = 253)**	**Control (*n* = 87)**	**Treatment (*n* = 166)**	***P*-value**
Age (years)	72 (68,78)	72 (68,79)	72 (68,77)	0.512
Sex (male)	157 (62.1)	55 (63.2)	102 (61.4)	0.892
**Underlying diseases**, ***n*** **(%)**				
Chronic obstructive pulmonary disease	19 (7.5)	7 (8)	12 (7.2)	0.806
Heart failure	92 (36.4)	39 (44.8)	53 (31.9)	0.054
Diabetic mellitus	74 (29.2)	25 (28.7)	49 (29.5)	1
Liver Cirrhosis	3 (1.2)	1 (1.1)	2 (1.2)	1
Tumor	44 (17.4)	10 (11.5)	34 (20.5)	0.082
Chronic renal failure	18 (7.1)	8 (9.2)	10 (6)	0.44
**Source of admission**, ***n*** **(%)**				
Internal medicine	40 (15.8)	16 (18.4)	24 (14.5)	0.469
Surgery	75 (29.6)	23 (26.4)	52 (31.3)	0.47
Emergency	62 (24.5)	18 (20.7)	44 (26.5)	0.357
Outside hospital	76 (30.0)	24 (27.6)	52 (31.3)	0.567
**Disease severity scores**				
APACHE II score	18 (16,22)	18 (15,21)	19 (16,22.25)	0.373
Sequential organ failure score	11.49 ± 3.95	11.23 ± 3.88	11.62 ± 4	0.457
Clinical pulmonary infection score	6 (5,8)	8 (6,9)	8 (6,9.3)	0.789
**Vital signs**				
Heart rate (beats/min)	99 (86.5,115.5)	101 (90,115)	98 (85,116)	0.38
Respiratory rate (breaths/min)	22 (17,27)	22 (17,26)	22 (18,27)	0.748
Mean artery pressure (mm Hg)	88.02 ± 12.64	88.32 ± 12.82	87.86 ± 12.58	0.781
**Ventilator parameters**				
Tide volume (ml)	420 (390,480)	430 (400,480)	410 (390,482.5)	0.192
Inhalation oxygen concentration (%)	0.35 (0.3,0.4)	0.35 (0.28,0.40)	0.35 (0.31,0.4)	0.064
**Artery blood gas**				
Lactic acid (mmol/L)	2 (1,2)	2 (1,2)	2 (1,2)	0.673
Arterial oxygen pressure (mm Hg)	95.7 (78.45,116.84)	94.76 (80.7,116.8)	96 (77.87,116.97)	0.706
Carbon dioxide partial pressure (mm Hg)	39 (35.7,43.45)	39 (35.6,42.1)	38.95 (35.85,44.03)	0.742
Oxygen index	284 (207.5,360.5)	287 (227,380)	277.5 (202.75,348.5)	0.203
**Clinical laboratory evaluation**				
Creatinine (umol/L)	86 (67.5,146)	107 (73,169)	81 (61.5,144.25)	0.086
Albumin level (g/L)	32 (29,35)	32 (28,35)	31.5 (29,34)	0.597
Total bilirubin (umol/L)	16.7 (14.1,24.35)	16.5 (13.4,24.2)	16.7 (14.38,24.73)	0.247
**Life-sustaining treatments**, ***n*** **(%)**				
Need for mechanism ventilation	238 (94.1)	83 (95.4)	155 (93.4)	0.589
Need for vasopressor	163 (64.4)	53 (60.9)	110 (66.3)	0.41
Need for RRT	53 (20.9)	14 (16.1)	39 (23.5)	0.195
**Coexisting pathogens**, ***n*** **(%)**				
Bacteria	216 (85.4)	78 (89.7)	138 (83.1)	0.192
Fungal	26 (10.3)	7 (8)	19 (11.4)	0.515
Else	5 (2)	0 (0)	5 (3)	0.168
**Drug therapy**, ***n*** **(%)**				
Antibiotics for GNB	189 (74.7)	64(73.6)	125 (75.3)	0.763
Antibiotics for GPB	237 (93.7)	83(95.4)	154 (92.8)	0.588
Antifungal drugs	85 (33.6)	31(35.6)	54 (32.5)	0.675
**Outcomes**				
Ventilation day (days)	5 (4,7)	5 (4,8)	5 (4,6)	0.045
ICU duration (days)	10 (5,19)	9 (6,20)	10 (4,18.25)	0.699
ICU mortality, *n* (%)	49 (19.4)	25 (28.7)	24 (14.5)	0.008
28 day mortality, *n* (%)	52 (20.6)	27 (31)	25 (15.1)	0.005

*Continuous variables are expressed as mean ± standard deviation or median (interquartile range). P-values reflect comparisons between the control and treatment groups. ICU, intensive care unit; APACHE II, Acute Physiology and Chronic Health Evaluation II; oxygen index, (partial pressure of oxygen) / (fraction of inspirated oxygen); RRT, continuous renal replacement therapy; GPB, Gram-positive bacteria; GNB, Gram-negative bacteria*.

There was no significant difference in the ICU duration between the groups. Patients in the treatment group had a significantly shorter duration of mechanical ventilation [5 (4, 6) vs. 5 (4, 8) days, *P* = 0.045], and a lower ICU mortality [14.5% (24/166) vs. 28.7% (25/87); *P* = 0.008] and 28 day mortality [15.1% (25/166) vs. 31% (27/87); *P* = 0.005] compared with those in the control group.

### Clinical Differences Between Survivors and Non-survivors

The clinical characteristics of all patients and for survivors and non-survivors (28 day) are shown in Table 2. Non-survivors had a higher APACHE II score, higher CPIS score, faster respiratory rate, higher carbon dioxide partial pressure on ICU admission, and longer mechanical ventilation compared with survivors (all *P* < 0.05). The rates of heart failure and chronic renal failure were significantly higher in non-survivors than in survivors (both *P* < 0.05). The requirement for renal replacement therapy was significantly more frequent in the in non-survivors compared with survivors (32.7 vs. 17.9%, *P* = 0.034). Importantly, more patients received nurse-led GDLPT in survivors than in non-survivors (70.1 vs. 48.1%, *P* = 0.005).

**Table 2:** Characteristics of the study population in survivors and non-survivors according to 28 day mortality.

**Variables**	**Sepsis (*n* = 253)**	**Non-Survival (*n* = 52)**	**Survival group (*n* = 201)**	***P*-value**
Age (years)	72 (68,78)	73 (69,78)	71 (68,78)	0.214
Sex (male)	157 (62.1)	34 (65.4)	123 (61.2)	0.633
**Underlying diseases**, ***n*** **(%)**				
Chronic obstructive pulmonary disease	19 (7.5)	7 (13.5)	12 (6.0)	0.079
Heart failure	92 (36.4)	28 (53.8)	64 (31.8)	0.006
Diabetic mellitus	74 (29.2)	20 (38.5)	54 (26.9)	0.124
Liver Cirrhosis	3 (1.2)	1 (1.9)	2 (1.0)	0.5
Tumor	44 (17.4)	13 (25.0)	31 (15.4)	0.149
Chronic renal failure	18 (7.1)	10 (19.2)	8 (4.0)	0.001
**Source of admission**, ***n*** **(%)**				
Internal medicine	40 (15.8)	6 (11.5)	34 (16.9)	0.348
Surgery	75 (29.6)	16 (30.8)	59 (29.4)	0.866
Emergency	62 (24.5)	12 (23.1)	50 (24.9)	0.858
Outside hospital	76 (30.0)	16 (30.8)	60 (29.9)	1
**Disease severity scores**				
APACHE II score	18 (16, 22)	21.5 (17, 26)	18 (15, 21)	0.001
Sequential organ failure score	11.49 ± 3.95	12.33 ± 3.82	11.27 ± 3.97	0.085
Clinical pulmonary infection score	6 (5, 8)	8 (6, 9)	6 (5, 8)	0.0001
**Vital signs**				
Heart rate (beats/min)	99 (86.5,115.5)	105 (90,119.75)	98 (85,114)	0.064
Respiratory rate (breaths/min)	22 (17,27)	24 (20,28)	21 (17,27)	0.039
Mean artery pressure (mm Hg)	88.02 ± 12.64	86.58 ± 13.63	88.39 ± 12.38	0.358
**Ventilator parameters**				
Tide volume (ml)	420 (390,480)	410 (392.5,480)	420 (390,490)	0.425
Inhalation oxygen concentration (%)	0.35 (0.3,0.4)	0.37 (0.32,0.42)	0.35 (0.3,0.4)	0.188
**Artery blood gas**				
Lactic acid (mmol/L)	2 (1, 2)	2 (1, 2)	2 (1, 2)	0.727
Arterial oxygen pressure (mm Hg)	95.7 (78.45,116.84)	103.35 (78.28,118.69)	93.31 (78.83,115.44)	0.125
Carbon dioxide partial pressure (mm Hg)	39 (35.7,43.45)	41 (36.25,46.23)	38.8 (35.5,42.85)	0.035
Oxygen index	284 (207.5,360.5)	282.5 (218.25,365.75)	284 (206.5,360.5)	0.952
**Clinical laboratory evaluation**				
Creatinine (umol/L)	86 (67.5,146)	114 (74.25,146.25)	83 (62,146)	0.103
Albumin level (g/L)	32 (29,35)	31 (29,33)	32 (28.5,35)	0.375
Total bilirubin (umol/L)	16.7 (14.1,24.35)	17.2 (12.73,40.63)	16.5 (14.2,23.5)	0.957
**Life-sustaining treatments**, ***n*** **(%)**				
Need for mechanism ventilation	238 (94.1)	51 (98.1)	187 (93)	0.319
Need for vasopressor	163 (64.4)	35 (67.3)	128 (63.7)	0.745
Need for RRT	53 (20.9)	17 (32.7)	36 (17.9)	0.034
Coexisting pathogens, *n* (%)
Bacteria	216 (85.4)	47 (90.4)	169 (84.1)	0.377
Fungal	26 (10.3)	7 (13.5)	19 (9.5)	0.442
Else	5 (2)	0 (0)	5 (2.5)	0.587
**Drug therapy**, ***n*** **(%)**				
Antibiotics for GNB	189 (74.7)	37 (71.2)	152 (75.6)	0.591
Antibiotics for GPB	237 (93.7)	51 (98.1)	186 (92.5)	0.205
Antifungal drugs	85 (33.6)	21 (40.4)	64 (31.8)	0.253
Received nurse-led GDLPT	166 (65.6)	25 (48.1)	141 (70.1)	0.005
**Outcomes**				
Mechanical ventilation days (days)	5 (4, 7)	5.5 (5, 12)	5 (4, 6)	0.0001
ICU duration (days)	10 (5, 19)	11 (7, 20)	9 (5, 19)	0.22

*Continuous variables are expressed as mean ± standard deviation or median (interquartile range). P-values reflect comparisons between the survivor and non-survivor groups. ICU, intensive care unit; APACHE II, Acute Physiology and Chronic Health Evaluation II; oxygen index, (partial pressure of oxygen) / (fraction of inspirated oxygen); RRT, continuous renal replacement therapy; GPB, Gram-positive bacteria; GNB, Gram-negative bacteria; GDLPT, goal-directed lung physiotherapy*.

### Risk Factors for 28 Day Mortality in Older Patients With Sepsis Caused by Pneumonia

Multivariate logistic regression analysis identified five factors that independently predicted 28-day mortality in patients with sepsis who were diagnosed with pneumonia. These factors were nurse-led GDLPT [odds ratio (OR), 0.379; 95% confidence interval (CI), 0.187–0.766; *P* = 0.007], heart failure (OR, 2.779; 95% CI, 1.360–5.679; *P* = 0.005), tumor (OR, 2.825; 95% CI, 1.181–6.757; *P* = 0.02), need for renal replacement therapy (OR, 2.450; 95% CI, 1.122–5.351; *P* = 0.025), and mechanical ventilation days (OR, 1.147; 95% CI, 1.055–1.247; *P* = 0.001). GDLPT was a protective factor for 28 day mortality (Table 3).

**Table 3:** Multivariate logistic regression analysis for predicting 28 day mortality in older patients with sepsis and pneumonia.

**Parameters**	**OR**	**95% CI**	***p*-value**
Heart failure	2.779	1.360–5.679	0.005
Tumor	2.825	1.181–6.757	0.020
Inhalation oxygen concentration	2.438	0.094–63.291	0.592
Creatinine	0.999	0.995–1.003	0.564
Need for continuous renal replacement therapy	2.450	1.122–5.351	0.025
Received nurse-led GDLPT	0.379	0.187–0.766	0.007
Mechanical ventilation days	1.147	1.055–1.247	0.001

*GDLPT, goal-directed lung physical therapy; OR, odds ratio; CI, confidence interval*.

## Discussion

Pneumonia is frequently encountered in older patients admitted to the ICU, with an incidence rate of >60% in those with sepsis (6). In numerous clinical studies, lung physiotherapy was analyzed in patients with various critical diseases (12, 13, 19). However, little is known about nurses providing lung physiotherapy to older patients with sepsis and pneumonia. Furthermore, the effect of lung physiotherapy on the prognosis of these patients is unclear. In this study, we found that nurse-led GLDPT was associated with shorter ventilation days, and reduced ICU mortality and 28 day mortality rates.

With aging, there are significant changes in the anatomical and physiological function of the lungs. Older people are more likely to develop pulmonary complications and underlying diseases, including COPD, aspiration, pneumonia, tumor, heart failure, chronic renal failure, and have a poor prognosis (14). The GDLPT protocol has several advantages over conventional physiotherapy. One advantage is that the frequency of oral care is determined by the Beck Oral Assessment Scale score and mucosal-plaque score. Another advantage is that enhancement of airway drainage is conducted every 4 h for 20 to 30 min each time, with a vibration frequency of 20 to 30 Hz. Additionally, the cough strength is evaluated every 6 h. Older people with a weak cough are more likely to have nosocomial pneumonia and aspiration pneumonia (15). In the USA, the incidence of aspiration pneumonia in older patients is 30.9 cases per 10,000 people, which is twice that in patients aged <65 years (16). A further advantage is that airway humidification is evaluated regularly to maintain humidification at degree II (17). For patients with specific pulmonary consolidation, goal-directed patient positioning is actively used. The frequency of turning over is adjusted, and a lung recruitment maneuver is also performed through mechanical ventilation or a manual resuscitator. Therefore, as a non-invasive intervention strategy, nurse-led GDLPT is useful for older patients with pneumonia.

In this nurse-led GDLPT study, pain assessment, delirium assessment, and active early activities were carried out every 6 h by nurses. Delirium varies from 11 to 42% in older patients and has adverse outcomes and high health care costs (18). Up to 60% of delirious patients were unrecognized unless actively screened, and sedative medications and deeper sedation are associated with the development of delirium (20). According to the patient's delirium status, early activities were carried out, such as sitting at the wheel and early walking. Frailty in older patients is also a risk of a longer ICU stay, prolonged ventilation duration, and increased mortality. Early exercise is the most effective intervention for debilitating syndrome and significantly improves muscle strength. In this nurse-led GDLPT study, old patients without delirium were able to get out of bed into a wheelchair early or walk early after therapy. Additionally, patients with intermittent delirium were able to perform bedside wheelchair activity, and patients with continuous delirium were able to receive passive movement in bed or sitting upon the bed. The nurse-led GDLPT protocol was formulated in the critical clinical setting and designed using more than 20 years of clinical experience, with specific items and strong operability. This therapy enabled good management of older patients.

In this study, nurse-led GDLPT significantly shortened the duration of ventilation. Prolonged mechanical ventilation might result in an increased incidence of ventilator-associated pneumonia, which is clinically meaningful, especially for older patients with pneumonia. A high frequency of antibiotic use and antibiotic resistance in patients is caused by ventilator-associated pneumonia. The prevalence of multidrug resistance is increasing, and ventilator-associated pneumonia caused by multidrug resistance is often fatal in the ICU (21). Several factors such as the CPIS score, and the APACHE II score contribute to a high percentage of multidrug resistance in the ICU and are important reasons for the poor prognosis of older patients with sepsis and pneumonia (22).

At present, older patients with a critical illness are frequently admitted in the ICU. However, data on older patients with pneumonia are relatively rare, and there have been few evidence-based medical reports (23). This study was a retrospective analysis based on data from a previous nurse-led GDLPT study. All collected data, including vital signs, ventilatory parameters, and laboratory indicators, were obtained from electronic information systems. Data were double checked to minimize the risk of data entry errors and there were missing data. Therefore, prospective, randomized, controlled trials are required to further investigate the role of nurse-led GDLPT on older patients with pneumonia. The nurse-led GDLPT study was a prospective, before-and-after study in a single center. Therefore, a multicenter study needs to be performed to further examine the details of GDLPT and validate its wide applicability in older patients with pneumonia.

## Conclusions

Managing pneumonia in critically ill older patients is a complex issue. Aging, comorbidities, frailty, and other factors in older patients significantly increase the management requirements and risks for those with critical illness. Nurse-led GDLPT significantly shortens the duration of ventilation and improves the 28 day mortality rate in older patients with sepsis and pneumonia. Nurse-led GDLPT is a new clinical intervention for the refined management of older patients with pneumonia, and it promotes the recovery of older patients with severe pneumonia.

## Data Availability Statement

The original contributions presented in the study are included in the article/Supplementary Material, further inquiries can be directed to the corresponding authors.

## Ethics Statement

The studies involving human participants were reviewed and approved by the Institutional Review Board of Peking Union Medical College Hospital (No. JS-1170). The study was registered at chictr.org.cn (identifier: ChiCTR1900025850). The patients/participants provided their written informed consent to participate in this study.

## Author Contributions

NC, QL, HW, and JS contributed to the conception of the study, data interpretation, and drafted the manuscript. WH, HL, and MZ contributed to the data collection and data analysis. WC and ZL contributed to data collection and interpretation and critically revised the manuscript for important intellectual content. All authors approved the final version of the manuscript.

## Funding

The work was supported by National Natural Science Foundation of China (No. 82072226), Beijing Municipal Science and Technology Commission (No. Z201100005520049), Fundamental Research Funds for the Central Universities (No. 3332021018), Non-profit Central Research Institute Fund of Chinese Academy of Medical Sciences (No. 2019XK320040), Tibet Natural Science Foundation (No. XZ2019ZR-ZY12(Z)), and Excellence Program of Key Clinical Specialty of Beijing in 2020 (No. ZK128001).

## Conflict of Interest

The authors declare that the research was conducted in the absence of any commercial or financial relationships that could be construed as a potential conflict of interest.

## Publisher's Note

All claims expressed in this article are solely those of the authors and do not necessarily represent those of their affiliated organizations, or those of the publisher, the editors and the reviewers. Any product that may be evaluated in this article, or claim that may be made by its manufacturer, is not guaranteed or endorsed by the publisher.
