# Supplementary material for: Implementation of Nurse-Led, Goal-Directed Lung Physiotherapy for Older Patients With Sepsis and Pneumonia in the ICU
Source: Front Med (Lausanne). 2021 Nov 22;8:753620. doi: 10.3389/fmed.2021.753620 (PMC8647879; doi:10.3389/fmed.2021.753620)
Supplement: Supplementary file 1 [file Table_1.DOCX]

**Supplementary file**

**Table 1 The protocols for pneumonia in the Control group (phase 1) and Treatment group (phase 2).**

| **The Control group** |
| --- |
| 1. Elevation of head of bed (30°–45°). |
| 2. Evaluation of the depth of sedation daily, with adjustment of sedative dose to maintain the Richmond agitation-sedation score (RASS) at −2 to 1[1]. |
| 3. Routine mouth care with chlorhexidine three times a day. |
| 4. Sputum aspiration as needed or every 2 h. |
| 5. Use of subglottic secretion drainage continuously. |
| 6. Routine auscultation. If abnormal results were found on auscultation, such as reduced breathing sound or bilateral asymmetric breathing sound, a bedside chest radiograph was conducted and the on-call doctor was informed. |
|  |
| **The treatment group** |
| 1. Elevation of head of bed (30°–45°). |
| 2. Evaluation of the depth of sedation daily, with adjustment of sedative dose to maintain the RASS at −2 to 1[1]. Delirium was assessed every 6 h by the Confusion Assessment Method for ICU (CAM-ICU), with early mobilization implemented as needed. Early mobilization included daily exercise programs tailored for individual patients, beginning with passive range of motion for unconscious patients, followed by active range of motion, bed activities, sitting up and moving on bed, and eventually walking. |
| 3. Routine mouth care with chlorhexidine three times a day, and was evaluated by the modified Beck Oral Assessment Scale (BOAS) score[2] and mucosal-plaque score[3]. The frequency of oral care was determined by the BOAS score, but was performed at least every 12 h. |
| 4. Airway drainage enhancement with a vibratory sputum extractor. This was performed every 4 h for 20–30 min each time, with a vibration frequency of 20–30 Hz. Cough intensity was assessed every 6 h to promote sputum drainage. The degrees of temperature and humidity were adjusted according to the sputum properties after sputum aspiration, to achieve sputum viscosity of grade II (moderate adhesion: following suction, a small quantity of sputum is retained on the inner wall of the tube, but is easily removed by washing with water)[4]. |
| 5. Use of subglottic secretion drainage continuously. |
| 6. Routine auscultation. If abnormal results were found on auscultation, such as reduced breathing sound or bilateral asymmetric breathing sound, lung ultrasound was immediately performed. If the ultrasound image indicated lung involvement such as tissue sample, debris sign, or air bronchi, the airway drainage was enhanced in the high lateral decubitus position (left side or right side: ≥90°) and the position was changed every 2 h. |
| 7. Performance of lung recruitment maneuver. Mechanical ventilation with continuous positive airway pressure (CPAP) of 35–40 cmH2O (1 cmH2O=0.098 kPa) was performed for 40 s once every 6–8 h. Only those who did not receive mechanical ventilation with an artificial airway were given dilation treatment, comprising artificial ventilation by adult manual resuscitators. Inhalation was deep and slow, followed by a pause of 15 s and then rapid exhalation. |

**References**

[1] Vincent JL (2017). Optimizing sedation in the ICU: the eCASH concept. Signa Vitae, 13:10-13.

[2] Beck S (1979). Impact of a systematic oral care protocol on stomatitis after chemotherapy. Cancer Nurs, 2:185-199.

[3] Henriksen BM, Ambjørnsen E, Axéll TE (1999). Evaluation of a mucosal-plaque index (MPS) designed to assess oral care in groups of elderly. Spec Care Dentist, 19:154-157.

[4] Yang M, Song Y, Pan L, Xie X (2019). Evaluation of the effect of two active warming and humidifying high-flow oxygen therapy systems in patients with tracheotomy. Biomed Rep, 11:31-37.
